# Integrative network toxicology and molecular docking reveal epigenetic and endocrine-disrupting mechanisms of PFAS in ovarian cancer

**DOI:** 10.3389/fphar.2026.1905013

**Published:** 2026-08-28

**Authors:** Yulu Wang, Xueli Liu, Yanan Ding, Zhanjie Zhao, Haotian Chen, Xiaojuan Wu, Yanzhou Yang, Pengge Pan, Luyi Li, Hui Gao

**Affiliations:** 1 Key Laboratory of Fertility Preservation and Maintenance of Ministry of Education, School of Basic Medicine, Ningxia Medical University, Yinchuan, China; 2 The First Clinical Medical College, Ningxia Medical University, Yinchuan, China; 3 Shengzhou Food and Drug Testing Center, Shaoxing, China; 4 Reproductive Medicine Center, The First Clinical Medical College, Ningbo University, Ningbo, China

**Keywords:** carcinogenicity, emerging PFAS, network toxicology, ovarian cancer, PFAS

## Abstract

**Objective:**

Per- and polyfluoroalkyl substances (PFAS) are omnipresent, persistent contaminants associated with rising ovarian cancer incidence; however, the molecular circuitry linking PFAS to ovarian tumorigenesis remains obscure.

**Methods:**

Here, an integrated network toxicology–molecular docking pipeline was developed to interrogate ten environmentally abundant PFAS.

**Results:**

Comprehensive interactome mapping identified 1,042 PFAS–protein interactions that converge on eight transcriptional hubs (HDAC1, SIRT1, ESR1, RXRA, PTGS2, MMP9, HDAC2 and HDAC3), subsequently validated as significantly overexpressed in ovarian tumours versus normal tissue pathway enrichment indicated that these mediators synchronize epigenetic silencing, estrogen signalling, inflammatory prostaglandin synthesis and extracellular-matrix remodelling, thereby may coordinate processes potentially associated with PFAS-associated initiation and progression. Competitive docking further showed that long-chain PFOS and PFOA are predicted the strongest binding affinities to the catalytic domains of HDACs 1–3, mechanistically explaining the observed transcriptomic alterations. Human ovarian granulosa-like tumor cell line (KGN) was exposed to PFOA, which works as a representative PFAS compound, showed a significant increase of cell proliferation and migration, leading to an alteration of gene expression previously identified by network toxicology.

**Discussion:**

Our findings provides a systems-level predictive framework for understanding PFAS-associated ovarian oncogenesis, furnish quantitative biomarkers for exposure–response modelling, and prioritize HDAC inhibition as prioritized candidate mediators to mitigate PFAS-related cancer risk with immediate translational implications for public-health protection and regulatory guideline revision.

## 1 Introduction

Per- and polyfluoroalkyl substances (PFASs) constitute a class of chemical compounds characterized by the substitution of hydrogen atoms bonded to carbon atoms with fluorine atoms in hydrocarbons (Evich et al., 2022; Zahmatkesh et al., 2024). As defined by the Organization for Economic Cooperation and Development (OECD), PFAS encompasses substances containing at least one fully fluorinated methyl (–CF3) or methylene (–CF2–) carbon atom, which is not bonded to any hydrogen, chlorine, bromine, or iodine atom (Jane et al., 2022). This definition includes more than 10,000 distinct PFAS molecules. PFASs are noted for their exceptional chemical stability, surface activity, thermal stability, hydrophobicity, and oil repellency, which facilitate their extensive application across various sectors, including industrial manufacturing, consumer goods, food packaging, and firefighting foams (Renner, 2006; Cordner et al., 2021; Kim et al., 2021). The primary sources of PFAS emissions are industrial production, consumer product usage, and wastewater treatment processes (Buck et al., 2011). During industrial production, PFASs are utilized as raw materials or additives, and as these products are used and subsequently discarded, PFASs are released into the environment. In recent years, PFASs have garnered significant attention due to their pervasive presence in the environment, biota, and human populations. PFASs present in consumer products are gradually released into the atmosphere, aquatic systems, and terrestrial environments during their usage (Dhore and Murthy, 2021; Gravesen et al., 2023; Lenka et al., 2021). Insufficiently treated wastewater from sewage treatment processes serves as a significant conduit for PFAS introduction into the environment. Furthermore, landfills, aqueous film-forming foams, and metal electroplating activities are primary sources of PFAS contamination (Yan et al., 2024; Jiang et al., 2024). In the United States, PFASs are detectable in more than 97% of human serum samples (Zhang et al., 2023; Yi et al., 2023). Exposure to PFAS has been shown to impact physiological processes, including immunotoxicity, lipid accumulation, and the development of certain cancers (Ehrlich et al., 2023; Bline et al., 2024; Yang et al., 2023; Gou et al., 2024; Winquist et al., 2023; Zheng et al., 2024). Consequently, PFAS, the persistent environmental pollutant, is frequently detected across various environmental compartments, such as air, water, sediment, and biota, posing potential risks to both ecological and human health.

Ovarian cancer ranks among the most prevalent malignant tumors affecting women globally, with its incidence and mortality rates varying significantly across different regions and populations (Konstantinopoulos and Matulonis, 2023; Cho and Shih Ie, 2009). Despite recent decreases in the global incidence and mortality rates of ovarian cancer, projections indicate a significant increase in both by 2040, particularly in low- and middle-income countries (Armstrong et al., 2022; Schoutrop et al., 2022). This underscores the urgent need for comprehensive global strategies to address the challenges posed by ovarian cancer, with the aim of enhancing prevention, diagnosis, and treatment, thereby reducing its incidence and mortality rates. Emerging evidence implicates PFAS in the promotion of cancer development and progression. A case–control study conducted by Cathey et al. (2023) demonstrated significantly elevated blood PFAS levels in patients with ovarian cancer. Long-chain PFAS have been shown to disrupt follicular maturation via the PPARγ pathway (Evans et al., 2022), with benchmark dose modeling indicating that human exposure levels could result in ovarian defects (Ding et al., 2020; Clar et al., 2024; Pattarawat et al., 2025). Additionally, cellular experiments have indicated that PFAS is predicted to influence epithelial–mesenchymal transition (EMT) through the TGF-β pathway, a process directly linked to ovarian cancer metastasis (Zhong et al., 2024). Consequently, it is imperative to investigate the relationship between PFAS exposure and ovarian cancer risk and to elucidate the underlying carcinogenic mechanisms.

In this study, we examined 10 specific types of PFASs and used network toxicology in conjunction with molecular docking techniques to explore the potential effects of PFAS on ovarian cancer. Additionally, we identified key molecules involved in this process. The outcomes of this research contribute to a deeper understanding of the role of PFAS in the pathogenesis of ovarian cancer and provide a scientific basis for strategies aimed at mitigating PFAS contamination to reduce cancer risk, thereby carrying substantial implications for public health.

## 2 Materials and methods

### 2.1 Identification of PFAS

Information on the chemical structures and related molecular information for PFAS were obtained from the PubChem database (Kim et al., 2025) (https://pubchem.ncbi.nlm.nih.gov). Four types of PFAS were included (17 in total): perfluoroalkyl carboxylic acid (PFCA), perfluoroalkyl sulfonic acid (PFSA), precursor, and emerging PFAS.

### 2.2 Network toxicological analysis of PFAS

SMILES sequences obtained from the PubChem database were imported into both the ADMETLAB 3.0 platform (Fu et al., 2024) (https://admetlab3.scbdd.com) and the ProTox3 database (Banerjee et al., 2024) (https://tox.charite.de/protox3) to verify the carcinogenicity of these pollutants.

### 2.3 Acquisition of PFAS-related targets

Potential target genes for PFAS were retrieved from the SwissTargetPrediction database (Daina et al., 2019) (http://www.swisstargetprediction.ch/), the TargetNet database (Yao et al., 2016) (http://targetnet.scbdd.com), and the SEA database (Keiser et al., 2007) (https://sea.bkslab.org/), with the target organism set to *Homo sapiens*. The gene data from these three databases were merged and deduplicated separately; gene names were standardized using the UniProt database (UniProt, 2025) (https://www.uniprot.org/), and the final target gene set for PFAS was compiled. The default thresholds/cutoffs for the SwissTargetPrediction, TargetNet, and SEA databases were a probability score > 0, a binding affinity score > 0, and a maximum Tanimoto similarity ≥ 0.4, respectively. Related targets of 10 PFASs were collected separately, and then, the final target gene set for PFAS was compiled (181 genes in total).

### 2.4 Acquisition of ovarian cancer-related targets

Ovarian cancer-related genes were retrieved from both the GeneCards database (Stelzer et al., 2016) (https://www.genecards.org/) and the OMIM database (McKusick, 2007) (https://omim.org/), with the keyword “ovarian cancer.” The gene selection criteria were as follows: the top 50% of genes ranked by GeneCards score and all genes collected from OMIM were merged and deduplicated to obtain the final set of ovarian cancer-related genes.

### 2.5 The intersection of PFAS–ovarian cancer targets

A Venn diagram was used to screen for common potential targets between PFAS and ovarian cancer, and the intersection was considered the potential targets for PFAS. The results were visualized using the Cloud platform (https://www.majorbio.com/tools).

### 2.6 Core target screening and protein interaction network construction

The screened potential targets between PFAS and ovarian cancer were imported into the STRING database (Szklarczyk et al., 2023) (https://string-db.org/, Version: 12.0). *Homo sapiens* was designated as the species of interest, with a “high confidence (0.700)” set specified as the minimum required interaction score, and disconnected nodes were hidden in the network. Subsequently, the results collected from STRING were visualized using Cytoscape software (Shannon et al., 2003) (v3.8.2), which constructed a protein–protein interaction (PPI) network with the core targets selected according to the degree values. The plugin of MCODE was used to identify the most significant subclusters of interacting nodes, and the plugin of CytoHubba was used to predict the top 10 significant genes using the maximal clique centrality (MCC) algorithm, which is one of the most robust algorithms for identifying biologically significant hubs in protein interaction networks.

### 2.7 GO and KEGG enrichment analyses

To clarify the metabolic disease pathways involved in the potential targets, the DAVID database (Sherman et al., 2022) (https://david.ncifcrf.gov/), developed by Kanehisa Laboratories, was used for GO term (biological processes, molecular functions, and cellular components) and KEGG pathway analyses. Visual analysis was conducted to effectively present the results of the GO and KEGG analyses (https://cnsknowall.com/).

### 2.8 Molecular docking analysis

Molecular docking provided an opportunity to analyze the intermolecular interactions between PFAS and their targets. PDB files for the key targets were retrieved from the Protein Data Bank, with *Homo sapiens* as the species of interest, and molecular structure files for PFAS were obtained from the PubChem database (https://pubchem.ncbi.nlm.nih.gov). After uploading both receptor (protein) and ligand (PFAS) structures, we performed molecular docking analysis using the CB-Dock2 website (Liu et al., 2022) (https://cadd.labshare.cn/cb-dock2/php/blinddock.php#job_list_load). Sequential docking analysis was performed, and the binding free energy scores were obtained, where lower free energy values correspond to more stable binding configurations.

### 2.9 Clinical relevance and prognosis analysis

The expression profile of the key potential genes across OV samples and paired normal tissues was analyzed using the GEPIA database (Tang et al., 2017) (http://gepia.cancer-pku.cn/index.html). The Kaplan–Meier Plotter (Győrffy, 2024) (https://www.kmplot.com/analysis/) was used to evaluate the prognostic value of OV, including overall survival (OS), progression-free survival (PFS), and post-progression survival (PPS). Mutation frequencies and copy number variations of the key potential genes in ovarian cancer were analyzed using the cBioPortal online resource (Gao et al., 2013) (https://www.cbioportal.org/), examining the correlation between genetic alterations and OV prognosis.

### 2.10 CCK8 assay

KGN cells were seeded onto 96-well plates. The cells were treated with DMSO-dissolved perfluorooctanoic acid (PFOA) for 24 h, 48 h, or 72 h after adherence. After treatment, the CCK 8 reagent solution was added to each well and incubated in the dark, and the OD value was determined at 450 nm using a microplate reader (Thermo Fisher, Multiskan Go, United States). The assay was performed in three independent biological replicates, each with three technical replicates (n = 3). Results are presented as mean ± standard deviation (SD). Two-way ANOVA with Dunnett’s *post hoc* test for multiple comparisons was used for statistical analyses.

### 2.11 Colony formation assay

KGN cells were seeded onto 6-well plates and treated with PFOA (1 μM) after adherence. Then, cells were incubated at 37 °C in a CO_2_ incubator. After 12 days, the cells were washed with PBS, fixed with 4% formaldehyde, and stained with 0.1% crystal violet. The colony number was counted under a microscope in 10 random fields. The assay was performed in three independent biological replicates, each with three technical replicates (n = 3). Results are presented as mean ± SD. An unpaired two-tailed t-test was used for statistical analyses.

### 2.12 Wound healing assay

KGN cells were seeded onto 6-well plates, and a wound was created using a sterile 200-µl pipette tip when the cells reached 70% confluence. Cells were then cultured in fresh serum-free medium treated with/without PFOA (1 μM) for 24 h. The images of the wounds were observed at 0 and 24 h under a bright-field microscope, and the widths of wounds were quantified. The assay was performed in three independent biological replicates, each with three technical replicates (n = 3). Results are presented as mean ± SD. An unpaired two-tailed t-test was used for statistical analyses.

### 2.13 RNA isolation and RT-PCR

Total RNA was extracted using TRIzol reagent according to the manufacturer’s protocol. For detection of HDAC1 (forward: 5′-GAA​CTG​GGG​ACC​TAC​GGG-3'; reverse: 5′-GCT​CTT​GAC​AAA​TTC​CAC​ACA​C-3′), SIRT1 (forward: 5′-GCT​GAC​GAC​TTC​GAC​GAC​G-3'; reverse: 5′-TCG​GTC​AAC​AGG​AGG​TTG​TCT-3′), ESR1 (forward: 5′-CCT​TCT​AGA​CCC​TTC​AGT​GAA​GCC-3'; reverse: 5′-CGA​GAC​CAA​TCA​TCA​GAA​TCT​CC-3′), RXRA (forward: 5′-TTC​TCT​ACC​CAG​GTG​AAC​TC-3'; reverse: 5′-AGG​AGG​CCA​TAT​TTC​CTG​AG-3′), PTGS2 (forward: 5′-TGT​TGA​AAA​GTA​GTT​CTG​GG-3'; reverse: 5′-AAG​CAG​GCT​AAT​ACT​GAT​AGG-3′), MMP9 (forward: 5′-GAA​GGC​AAA​CCC​TGT​GTG​TT-3'; reverse: 5′-AGA​GTA​CTG​CTT​GCC​CAG​GA-3′), HDAC2 (5′-AGC​ATC​AGG​ATT​CTG​TTA​CGT​TAA​TGA-3'; reverse: 5′-CAA​CAC​CAT​CAC​CAT​GAT​GAA​TAT​CT-3′), and HDAC3 (5′-TGA​TGA​CCA​GAG​TTA​CAA​GCA​C-3'; reverse: 5′-GGG​CAA​CAT​TTC​GGA​CAG-3′), isolated RNA was reversed into complementary DNA (cDNA), and a Bio-Rad PCR detection system (Bio-Rad, United States) was used for RT-PCR. The expression levels of eight abovementioned genes were normalized to that of GAPDH, which served as the internal control. The assay was performed in three independent biological replicates, each with three technical replicates (n = 3). Results are presented as mean ± SD. An unpaired two-tailed t-test was used for statistical analyses.

### 2.14 Statistical analysis

All statistical analyses were performed using R software (v4.1.0). Comparisons of gene expression between tumor and normal tissues were conducted using the Wilcoxon rank-sum test. Survival curves were compared using the log-rank test. A p-value < 0.05 was considered statistically significant.

## 3 Results

### 3.1 Toxicity assessment of PFAS

Toxicity assessments were conducted for PFAS. We divided the substances into four groups for PFAS family substances mainly according to the structure: PFCA (including PFBA, PFPeA, PFHxA, PFHpA, PFOA, PFNA, PFDA, PFUA, PFDoA, and PFTrDA), PFSA [including PFBS, PFHxS, and perfluorooctane sulfonate (PFOS)], precursor (including FOSA), and emerging PFAS (including 6:2 Cl-PFESA, 8:2 Cl-PFESA, and OBS). Therefore, there are 17 PFAS family substances evaluated for toxicity, and the full name, molecular formula, and SMILES structure are shown in Table 1.

**TABLE 1:** Full name, molecular formula, SMILES structure, and carcinogenicity of PFAS.

Acronym	Full name	Molecular formula	SMILES structures	Carcinogenicity(ADMETLAB3.0)	Carcinogenicity(ProTox3)
Perfluoroalkyl carboxylic acids (PFCAs)					
PFBA	Perfluorobutanoic acid	C_4_HF_7_O_2_	C(=O)(C(C(C(F)(F)F)(F)F)(F)F)O	0.400	0.63
PFPeA	Perfluoropentanoic acid	C_5_HF_9_O_2_	C(=O)(C(C(C(C(F)(F)F)(F)F)(F)F)(F)F)O	0.439	0.63
PFHxA	Perfluorohexanoic acid	C_6_HF_11_O_2_	C(=O)(C(C(C(C(C(F)(F)F)(F)F)(F)F)(F)F)(F)F)O	0.399	0.63
PFHpA	Perfluoroheptanoic acid	C_7_HF_13_O_2_	C(=O)(C(C(C(C(C(C(F)(F)F)(F)F)(F)F)(F)F)(F)F)(F)F)O	0.452	0.63
PFOA	Perfluorooctanoic acid	C_8_HF_15_O_2_	C(=O)(C(C(C(C(C(C(C(F)(F)F)(F)F)(F)F)(F)F)(F)F)(F)F)(F)F)O	0.454	0.63
PFNA	Perfluorononanoic acid	C_9_HF_17_O_2_	C(=O)(C(C(C(C(C(C(C(C(F)(F)F)(F)F)(F)F)(F)F)(F)F)(F)F)(F)F)(F)F)O	0.457	0.63
PFDA	Perfluorodecanoic acid	C_10_HF_19_O_2_	C(=O)(C(C(C(C(C(C(C(C(C(F)(F)F)(F)F)(F)F)(F)F)(F)F)(F)F)(F)F)(F)F)(F)F)O	0.452	0.63
PFUA	Perfluoroundecanoic acid	C_11_HF_21_O_2_	C(=O)(C(C(C(C(C(C(C(C(C(C(F)(F)F)(F)F)(F)F)(F)F)(F)F)(F)F)(F)F)(F)F)(F)F)(F)F)O	0.45	0.63
PFDoA	Perfluorododecanoic acid	C_12_HF_23_O_2_	C(=O)(C(C(C(C(C(C(C(C(C(C(C(F)(F)F)(F)F)(F)F)(F)F)(F)F)(F)F)(F)F)(F)F)(F)F)(F)F)(F)F)O	0.446	0.63
PFTrDA	Perfluorotridecanoic acid	C_13_HF_5_O_2_	C(=O)(C(C(C(C(C(C(C(C(C(C(C(C(F)(F)F)(F)F)(F)F)(F)F)(F)F)(F)F)(F)F)(F)F)(F)F)(F)F)(F)F)(F)F)O	0.441	0.63
Perfluoroalkyl sulfonic acids (PFSAs)					
PFBS	Perfluorobutane sulfonic acid	C_4_HF_9_O_3_S	C(C(C(F)(F)S(=O)(=O)O)(F)F)(C(F)(F)F)(F)F	0.438	0.32
PFHxS	Perfluorohexane sulfonic acid	C_6_HF_13_O_3_S	C(C(C(C(F)(F)S(=O)(=O)O)(F)F)(F)F)(C(C(F)(F)F)(F)F)(F)F	0.457	0.32
PFOS	Perfluorooctane sulfonic acid	C_8_HF_17_O_3_	C(C(C(F)(F)F)(F)F)(C(OC(C(OC(C(O)(F)F)(F)F)(F)F)(F)F)(F)F)(F)F	0.331	0.46
Precursor	​	​	​	​	​
FOSA	Perfluorooctane sulfonamide	C_8_H_2_F_17_NO_2_S	C(C(C(C(C(F)(F)S(=O)(=O)N)(F)F)(F)F)(F)F)(C(C(C(F)(F)F)(F)F)(F)F)(F)F	0.422	0.47
Emerging PFAS					
6:2 Cl-PFESA	6:2 Chlorinated polyfluorinated ether sulfonate	C_8_ClF_16_KO_4_S	C(C(C(C(F)(F)Cl)(F)F)(F)F)(C(C(OC(C(F)(F)S(=O)(=O)[O-])(F)F)(F)F)(F)F)(F)F.[K+]	0.408	0.27
8:2 Cl-PFESA	8:2 Cl-polyfluorinated ether sulfonate	C_10_HClF_20_O_4_S	C(C(C(C(C(F)(F)Cl)(F)F)(F)F)(F)F)(C(C(C(OC(C(F)(F)S(=O)(=O)O)(F)F)(F)F)(F)F)(F)F)(F)F	0.403	0.38
OBS	Sodium p-perfluorous nonenoxybenzene sulfonate	C_15_H_4_F_17_NaO_4_S	C1=CC(=CC=C1OC(=C(C(C(F)(F)F)(C(F)(F)F)F)C(C(F)(F)F)(C(F)(F)F)F)C(F)(F)F)S(=O)(=O)[O-].[Na+]	0.557	0.2

**FIGURE 1:**
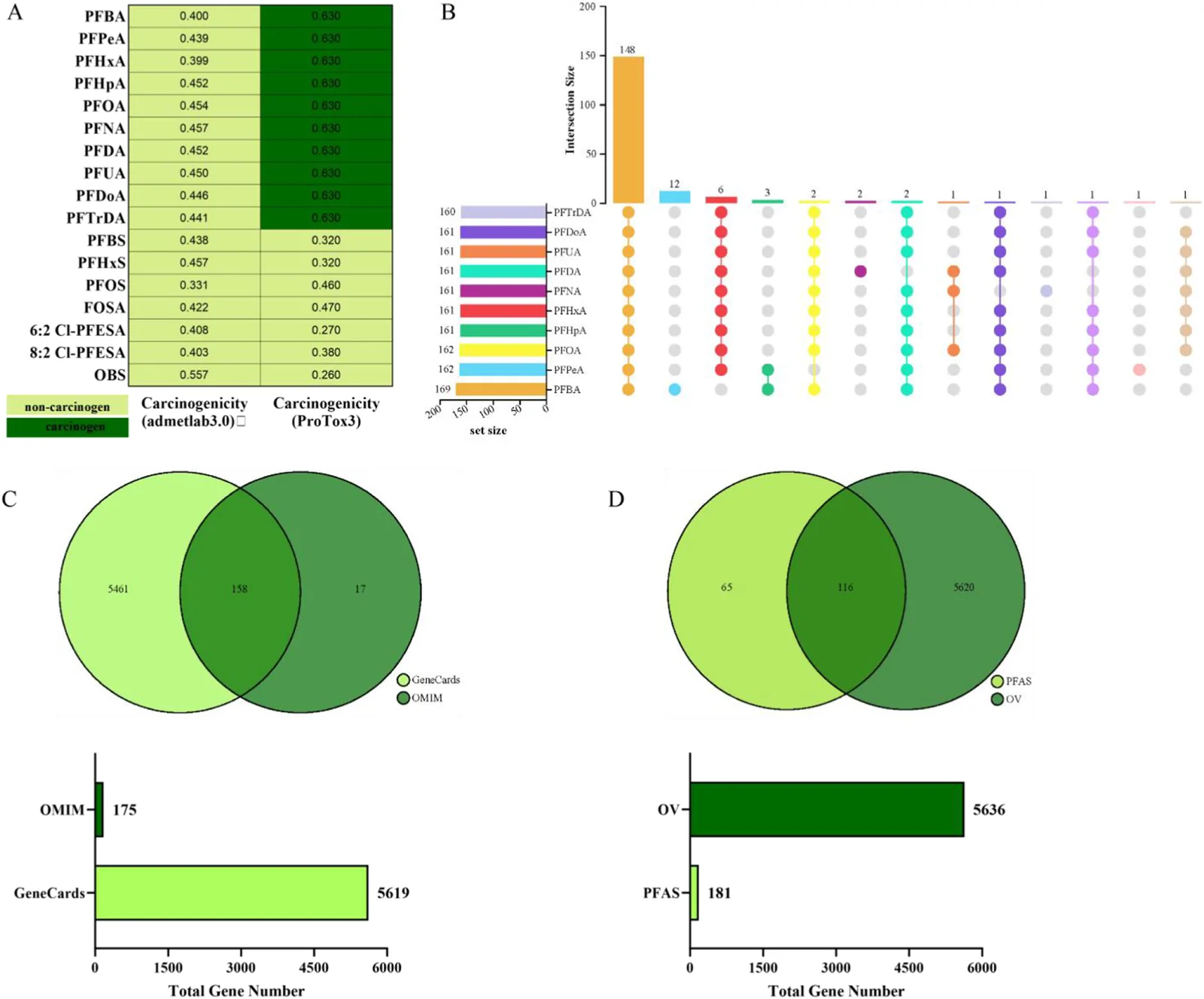
The impact of PFAS on ovarian cancer. **(A)** Carcinogenicity was evaluated using the ADMETLAB 3.0 and ProTox-3 databases; **(B)** the number of potential target genes corresponding to each PFAS with carcinogenicity after integrating the data obtained from the SwissTargetPrediction, TargetNet, and SEA databases; **(C)** target genes of ovarian cancer; **(D)** identification of PFAS–ovarian cancer target genes.

The ADMETLAB 3.0 platform and the ProTox3 database were used for the toxicity assessment of these PFASs. The toxicological predictions from both platforms were compiled systematically, as shown in Figure 1A, and the PFAS family substance was considered toxic if carcinogenicity was predicted on either platform. The results showed that 10 PFAS family substances showed carcinogenicity.

### 3.2 Target genes of PFAS

We obtained the number of potential target genes corresponding to each PFAS by integrating data from the SwissTargetPrediction, TargetNet, and SEA databases, and after merging these datasets, combining the targeted genes of each PFAS, and removing duplicates, we obtained a final collection of 181 unique PFAS targets. Among them, 148 target genes were shared by PFAS with carcinogenicity (Figure 1B).

### 3.3 Target genes of ovarian cancer

With the keyword “ovarian cancer,” we retrieved 11,237 target genes from the GeneCards database and 451 target genes from the OMIM database. After selecting the top 50% of genes ranked by GeneCards score and all genes from the OMIM database and removing duplicates, we identified a final set of 5,636 ovarian cancer-related genes. Among them, 158 target genes were shared by both the GeneCards and OMIM databases (Figure 1C).

### 3.4 Identification of PFAS–ovarian cancer target genes

Through integration of the datasets comprising 181 PFAS targets and 5,636 ovarian cancer-related genes using Venn analysis, we identified 116 overlapping genes (Figure 1D). We cross-referenced these predicted targets with known PFAS targets from the Comparative Toxicogenomics Database (CTD), and we found that 67 of our 181 predicted PFAS targets (37%) were documented in CTD as being associated with PFAS exposure, providing external validation of our prediction pipeline. These overlapping genes represented the potential targets through which PFAS may promote the occurrence and development of ovarian cancer. Notably, these interactions were derived from *in silico* target prediction databases and represented potential associations.

### 3.5 Interaction network construction and enrichment analysis

**FIGURE 2:**
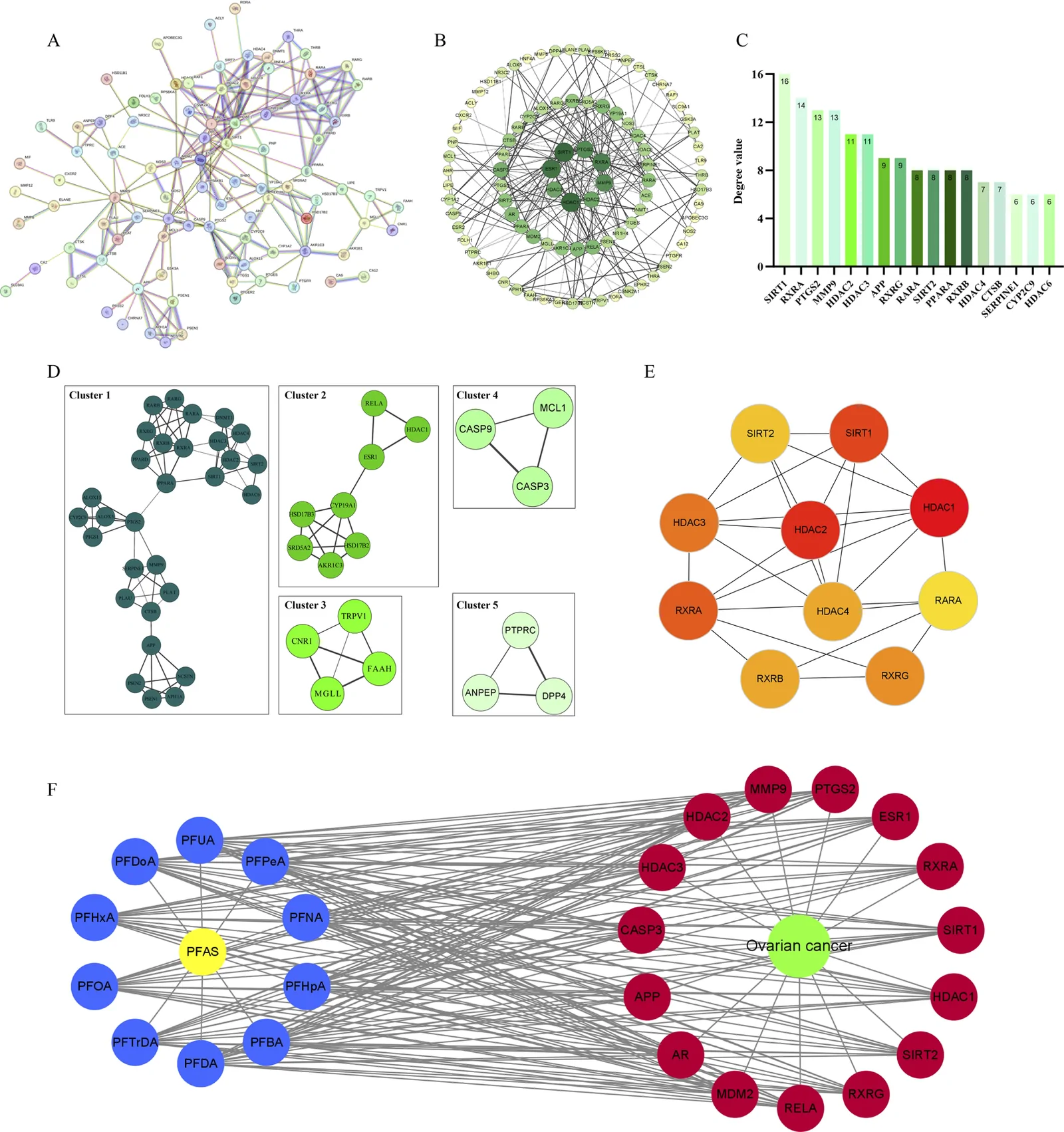
The associations between PFAS and ovarian cancer. **(A)** The PPI network constructed using the STRING database, which highlighted the functional associations of the targets. Edges indicate interactions, and nodes indicate proteins. **(B)** The PPI network of the potential targets analyzed using Cytoscape. Nodes are colored and sized according to the degree values, with darker colors and larger circles indicating stronger interactions. **(C)** List of targets with the degree value more than 5. **(D)** Mcode classification of core targets. **(E)** Top 10 Hubba gene of core targets. **(F)** PFAS–potential target–ovarian cancer network.

A total of 116 PFAS–ovarian cancer intersecting targets were imported into the STRING database to construct a PPI network with a confidence level of 0.7, and 93 PFAS–ovarian cancer-related targets remained after isolates were filtered out. In total, we collected 93 nodes and 4,294 edges for the network (Figure 2A). Subsequently, visualization of the PPI network for these PFAS–ovarian cancer-related targets was created to display the connections. As shown in Figure 2B, the darker the green and the larger the circle, the greater the representation value. The degree value above 5 is shown in Figure 2C. The MCODE plugin was installed to classify the disease targets associated with ovarian cancer, and the results showed that there were a total of five sub-network modules (Figure 2D). At the same time, the CytoHubba plugin was installed to identify the top 10 hub genes according to the MMC algorithm, namely, HDAC1, HDAC2, SIRT1, RXRA, HDAC3, RXRG, HDAC4, RXRB, SIRT2, and RARA (Figure 2E). Subsequently, a network model of “PFAS-intersecting tagets-ovarian cancer” was established using Cytoscape software: blue circle nodes represent PFAS family substances with carcinogenicity, green circle nodes represent ovarian cancer, red circle nodes represent intersecting targets, and gray connecting lines denote the interaction among PFAS, targets, and ovarian cancer (Figure 2F). Each of these genes is well documented in the literature as playing critical roles in processes relevant to both PFAS toxicity and ovarian cancer. For example, HDAC1/2/3 are associated with epigenetic regulation, histone deacetylation, and tumor suppressor silencing; SIRT1 is associated with NAD+ dependent deacetylase, oxidative stress response, and DNA repair; ESR1 is associated with endocrine disruption and hormone-driven carcinogenesis; RXRA is associated with PPAR heterodimer partner and metabolic regulation; PTGS2 is associated with prostaglandin synthesis, inflammation, and tumor promotion; MMP9 is associated with extracellular matrix remodeling, invasion, and metastasis. These findings suggested that these targets may play significant roles in the prevention and treatment of PFAS-induced ovarian cancer.

### 3.6 GO and KEGG enrichment analyses for targets

**FIGURE 3:**
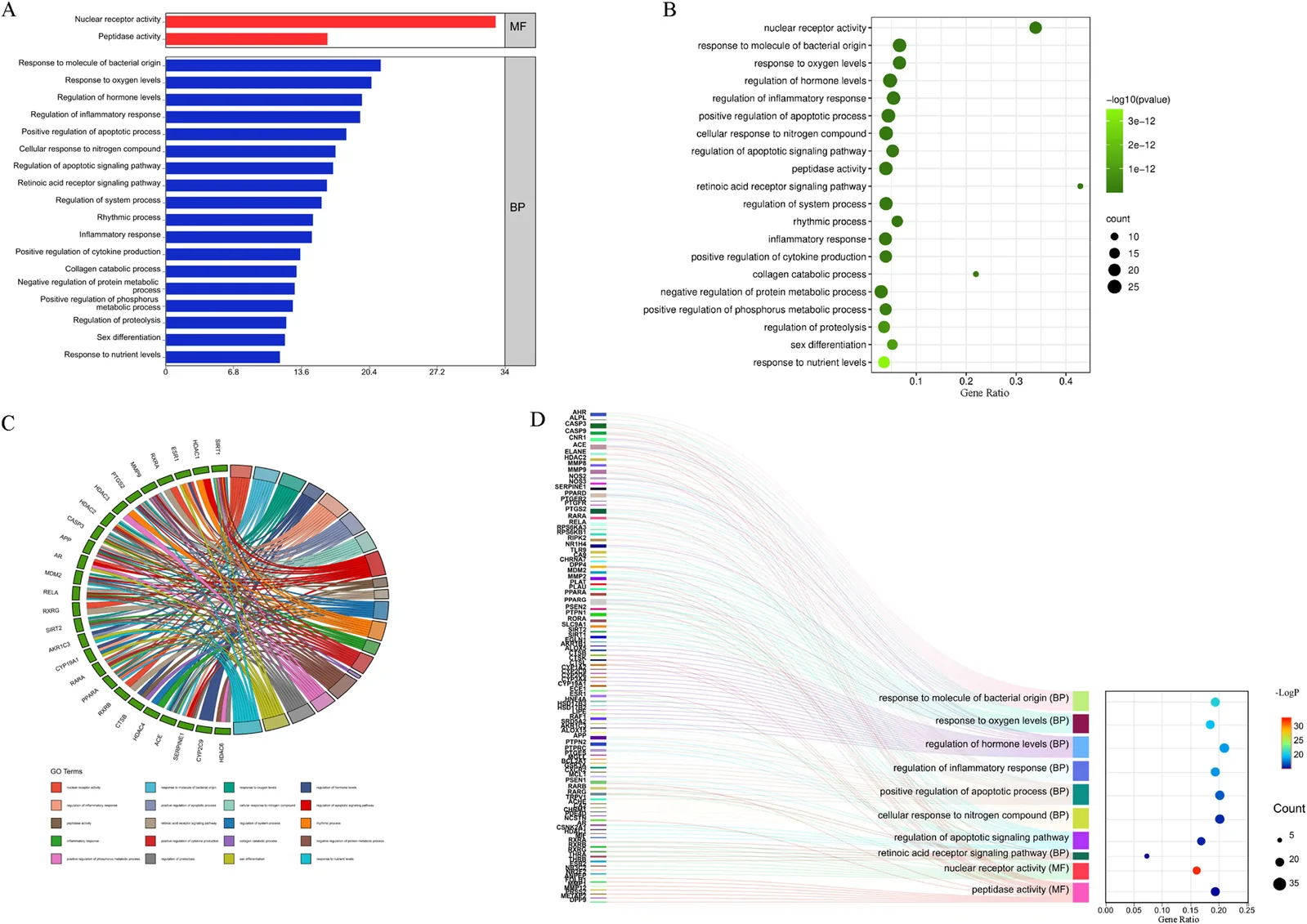
GO and KEGG pathway enrichment analyses of the potential targets. **(A)** GO enrichment analysis of PFAS–ovarian cancer targets. **(B)** Bubble map of GO enrichment analysis. **(C)** Circular chord diagram of KEGG enrichment analysis. **(D)** Sankey diagram of the top 10 KEGG enrichment results.

The targets were uploaded to the DAVID database for further analyses of GO and KEGG enrichment, specifically focusing on *Homo sapiens*. Then, we selected the top 20 GO terms determined using score values for visualization. As shown in Figures 3A,B, GO functional enrichment analysis illustrated that these genes are mainly involved in biological processes, such as response to oxygen levels, regulation of hormone levels, regulation of inflammatory response, and regulation of apoptotic process. Meanwhile, KEGG enrichment analysis was conducted to identify the participation of particular signaling pathways involved (Figure 3C). Figure 3D shows the top 10 KEGG pathways, among which response to oxygen levels, regulation of hormone levels, and other pathways are the most significant. These pathways represent predicted functional associations based on target gene membership, and their activation status under PFAS exposure has not been experimentally verified.

### 3.7 Molecular docking analysis

**FIGURE 4:**
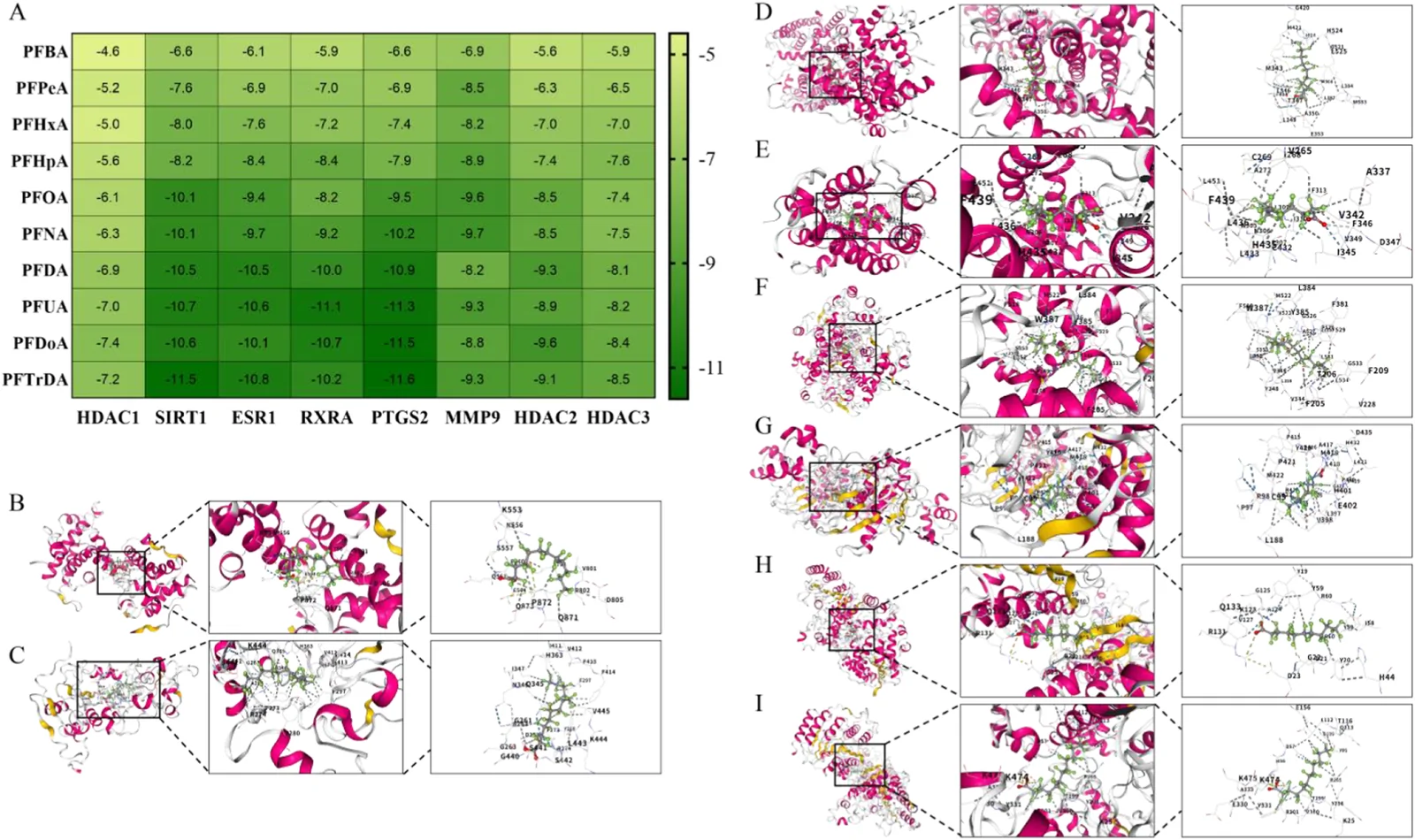
GO and KEGG pathway enrichment analyses of the potential targets. **(A)** The heatmap suggested the binding energies (kcal/mol) derived from molecular docking analysis between eight key molecules (HDAC1, SIRT1, ESR1, RXRA, PTGS2, MMP9, HDAC2, and HDAC3) and 10 PFAS family substances. **(B)** Molecular docking visualization of HDAC1 with PFDoA. **(C)** Molecular docking visualization of SIRT1 with PFTrDA. **(D)** Molecular docking visualization of ESR1 with PFDA. **(E)** Molecular docking visualization of RXRA with PFUA. **(F)** Molecular docking visualization of PTGS2 with PFTrDA. **(G)** Molecular docking visualization of MMP9 with PFNA. **(H)** Molecular docking visualization of HDAC2 with PFDA. **(I)** Molecular docking visualization of HDAC3 with PFDoA.

Molecular docking analysis was performed to analyze the affinity of 10 PFAS family substances with the potential targets. As shown in Figure 4A, all the PFAS family substances selected formed stable and spontaneous interactions with the targets with the binding energy less than −5 kcal/mol, suggesting thermodynamically favorable interactions. The docking details of PFAS with targets are shown in Figures 4B–I, and the results showed the site-specific binding properties which involved hydrophobic interactions, hydrogen bonding, and potential electrostatic interactions. Molecular docking analysis suggested that PFASs are predicted to bind to the catalytic domains of targets with favorable computed affinities, suggesting a potential mechanism that warrants experimental validation, potentially affecting biological processes related to ovarian cancer.

### 3.8 Clinical relevance and prognostic analysis

**FIGURE 5:**
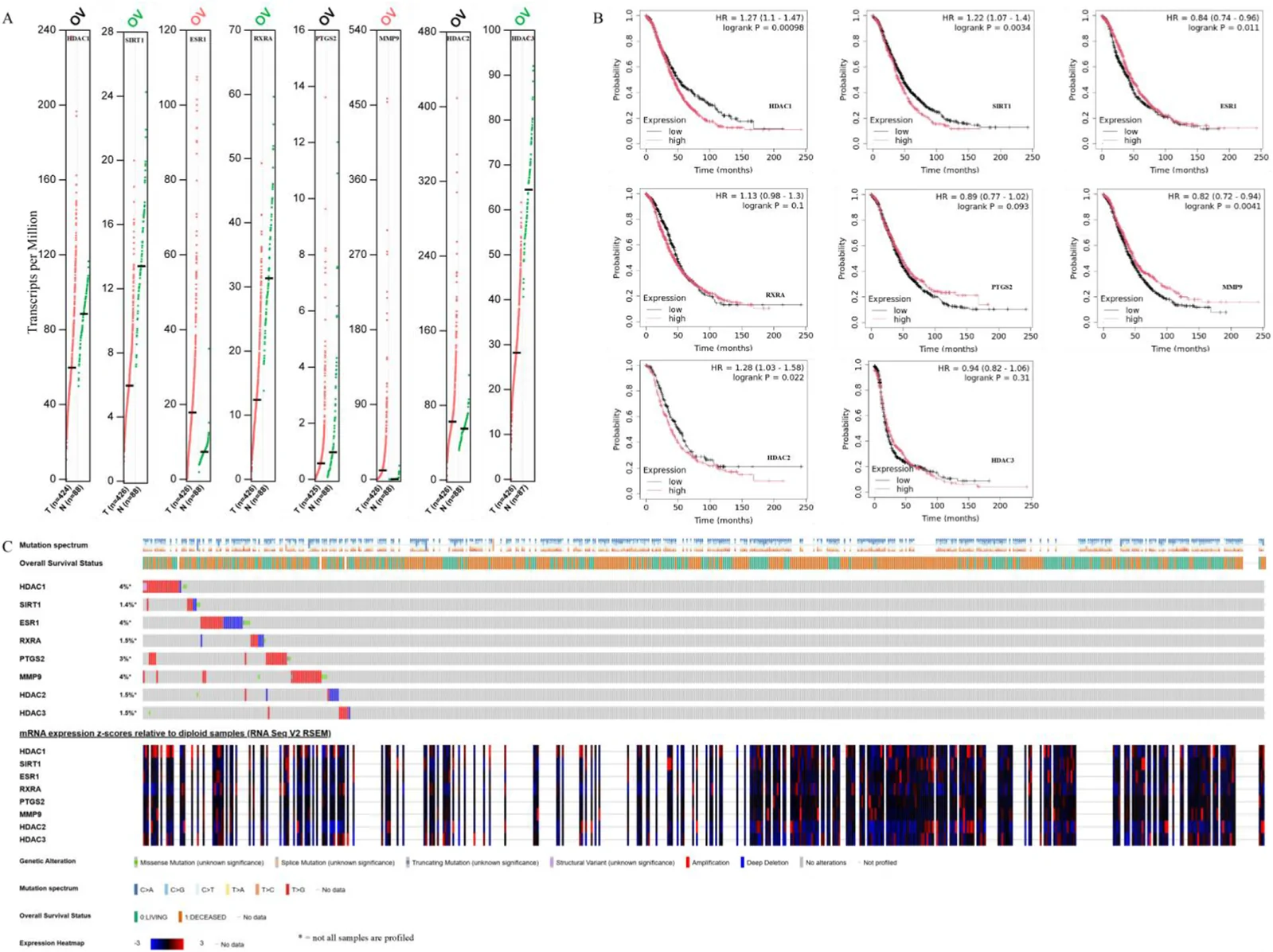
Clinical relevance and prognostic analysis of the potential targets. **(A)** The expression profile of targets across ovarian cancer and paired normal tissues plotted using the GEPIA database. **(B)** Overall survival curve of the potential targets in ovarian cancer. **(C)** The mutation spectrum, overall survival status, alteration frequency, and mRNA expression z-scores relative to diploid sample of the potential targets in ovarian cancer.

To further understand the relationship between ovarian cancer and targets, the expression profile of targets across ovarian cancer and paired normal tissues were plotted from the GEPIA database, facilitating a comparison of steady-state expression differences between ovarian cancer tumor tissues and corresponding normal tissues. Compared with paired normal tissues, ovarian cancer showed a significant increase in expression of ESR1 and MMP9 and a decrease in expression of SIRT1, RXRA, and HDAC3 (Figure 5A). Meanwhile, the Kaplan–Meier plotter database detecting OS was used for the identification of target expression in human ovarian cancer (Figure 5B). We found that high expression levels of HDAC1, SIRT1, and HDAC2 were associated with poorer OS in the KM Plotter database for ovarian cancer, whereas high expression levels of ESR1 and MMP9 were associated with better OS. Alterations of the targets in tumors from patients with ovarian cancers are shown in Figure 5C. A study (TCGA and PanCancer Atlas) on 585 patients (including 523 samples with mutations) was selected for analysis, and data showed that queried genes were altered in 109 (19%) of all the 585 queried patients/samples. The mutation spectrum, overall survival status, alteration frequency, and mRNA expression z-scores relative to the diploid sample of the targets in ovarian serous cystadenocarcinoma were plotted. These results indicated a robust link between the expression of targets and ovarian cancer pathology.

### 3.9 Effect of PFOA on proliferation, migration, and gene expression in the ovarian granulosa tumor cell line KGN

**FIGURE 6:**
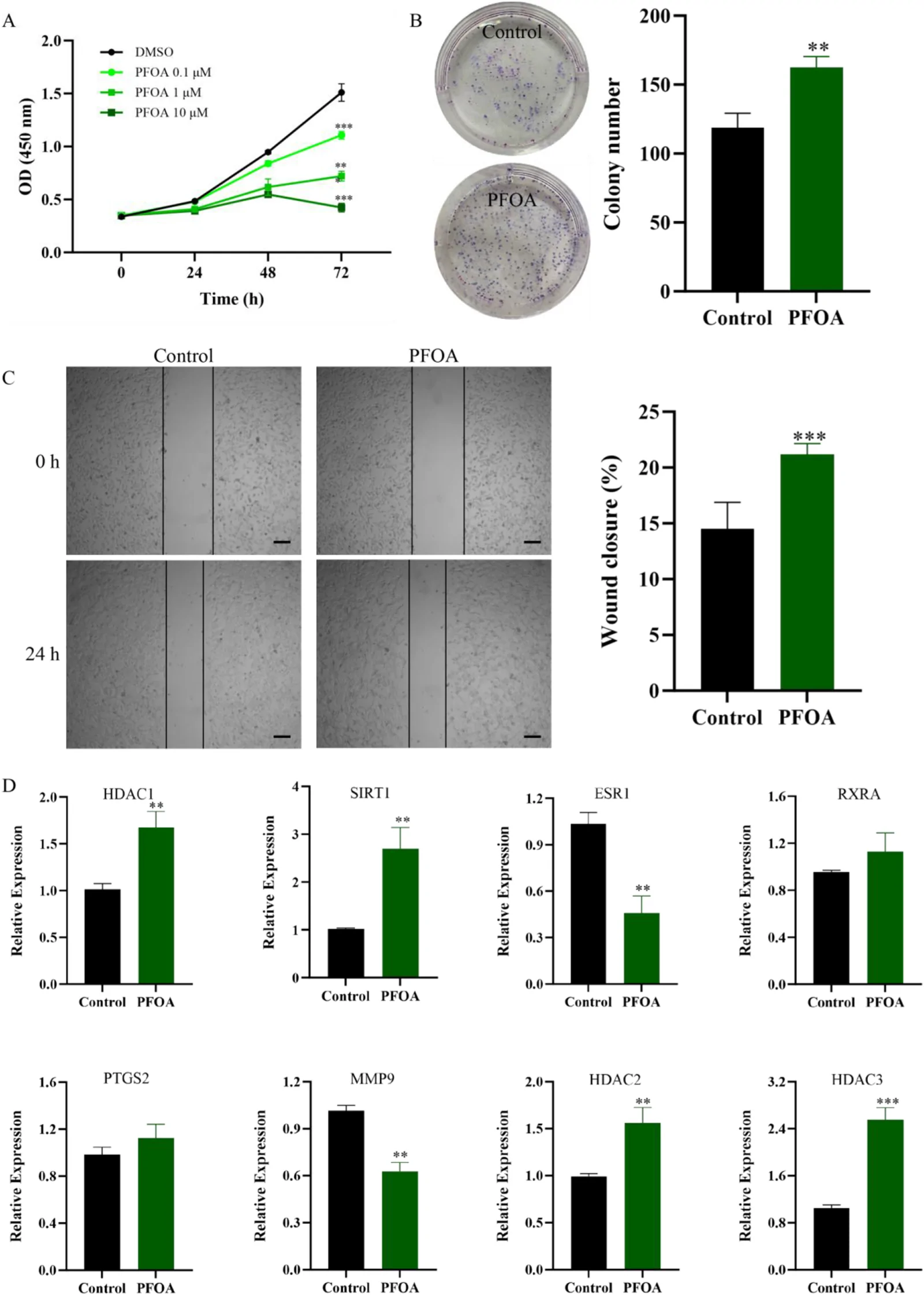
Effect of PFOA on proliferation, migration, and gene expression in the ovarian granulosa tumor cell line KGN. **(A)** Cell viability of KGN cells treated with PFOA of different concentrations (0, 0.1, 1, and 10 μM). **(B)** Colony formation assays were conducted after 48-h exposure to PFOA (1 μM). **(C)** The wound healing assay showed the widths of wounds following PFOA treatment. **(D)** RT-PCR was used to determine mRNA expression levels of HDAC1, SIRT1, ESR1, RXRA, PTGS2, MMP9, HDAC2, and HDAC3 following PFOA treatment.

As one of the PFAS family substances, PFOA was selected to examine the effects of PFAS on cell proliferation, migration, and gene expression in KGN. PFOA is one of the most abundant and extensively studied PFAS compounds, and conducting comprehensive cellular assays for all 10 compounds was beyond the scope of this initial study. PFOA concentrations (0.1, 1, and 10 µM) were selected to encompass environmentally relevant high-exposure levels (1 µM), occupational/reference levels (0.1 µM), and a supraphysiological concentration (10 µM) for mechanistic exploration, based on the prior literature reporting serum and follicular fluid PFAS levels. The CCK 8 assay indicated that KGN cells treated with DMSO (control) or different concentrations (0.1, 1, or 10 μM) of PFOA for 0–72 h induced time-dependent proliferation (Figure 6A). Colony formation assays were conducted after 48-h exposure to PFOA (1 μM), and data showed that the colony formation rate of KGN cells treated with 1 μM PFOA increased by 1.37-fold compared to the control group (Figure 6B). The wound healing assay showed that the widths of wounds of the PFOA-treated group was obviously narrowed compared with the control group at 24 h (Figure 6C). For the core targeted genes previously screened, we found four higher-expressed (HDAC1, SIRT1, HDAC2, and HDAC3) and two lower-expressed (ESR1 and MMP9) genes for PFOA-treated cells than in the control group (Figure 6D). Notably, mRNA expression changes reflect transcriptional responses; however, corresponding protein-level changes were not assessed, and mRNA up- or downregulation of these genes at 24 h does not predict its long-term functional effects or enzymatic activity. At the same time, these data were different from the data shown in Figure 5A; therefore, these two datasets were two distinct experimental systems, reflect different biological levels, and should be interpreted independently rather than directly compared. KGN cell experiments were conducted not to simply reproduce the expression patterns observed in clinical tissues, but rather to validate whether the target genes predicted by network toxicology exhibit transcriptional responses to direct PFOA exposure. Thus, PFOA altered the proliferation, migration, and gene expression in the ovarian granulosa tumor cell line KGN.

## 4 Discussion

Ovarian cancer, an age-associated malignancy, ranks as the eighth most prevalent cancer among women globally. The incidence of ovarian cancer is influenced by genetic predispositions, with carriers of BRCA1/2 mutations exhibiting an elevated risk ranging from 23% to 54% (Pujade-Lauraine et al., 2017). Epidemiological studies have identified infertility, persistent ovulation, and estrogen exposure as significant risk factors, whereas pregnancy, breastfeeding, and the use of oral contraceptives confer protective effects (Lheureux et al., 2019). Notably, the endocrine-disrupting properties of PFAS may interact with hormonal pathways, potentially disrupting the follicular microenvironment. For instance, PFAS can bind to the estrogen receptor (ERα) and activate oncogenic pathways such as PI3K/AKT/mTOR (Villeneuve et al., 2023; Bali et al., 2024; Young et al., 2021). Clinical investigations have also suggested that concentrations of PFOA and PFDA in follicular fluid are inversely correlated with embryo quality, indicating a potential impact on ovarian function through the dysregulation of sphingolipid metabolism. A meta-analysis conducted in 2025 further corroborated that exposure to mixed PFAS significantly increases the risk of ovarian cancer (OR = 1.63, 95% CI: 1.49–1.78) (Hallberg et al., 2023; Hong et al., 2022). Although the incidence rate in White women is higher than that in African Americans, it remains to be determined whether differences in the geographical distribution of PFAS exposure, such as between industrial and agricultural areas, contribute to this disparity, and this requires verification through environmental monitoring data.

Network toxicology offers insights into the ovarian toxicity mechanisms of PFAS by constructing a multi-level network encompassing “toxic components-biological targets-effect pathways.” By integrating databases such as SwissTargetPrediction and GeneCards, overlapping genes between PFAS and ovarian cancer were identified. A PPI network was constructed using tools such as STRING and Cytoscape, indicating key pathways regulated by PFAS-related ovarian cancer targets. This approach addresses the limitations of traditional toxicology in managing mixed effects. The core methodologies and technical framework of network toxicology emphasize systematic, multi-scale, and data-driven analyses, aiming to comprehensively understand the toxicity mechanisms of chemical substances and provide a scientific basis for toxicity assessment and risk management (Nogales et al., 2022). In future research, it is feasible to integrate single-cell sequencing data to analyze the specific targets of PFAS within ovarian cell subpopulations, such as granulosa cells and epithelial cells, thereby constructing a dynamic interaction network. Concurrently, the integration of transcriptomic and metabolomic data can enhance predictive accuracy, facilitating multi-omics validation.

PFASs have the potential to influence the onset, development, and progression of ovarian cancer through the endocrine disruption axis (Hammarstrand et al., 2021; Liu et al., 2024). These substances can mimic estrogen, activate estrogen receptor alpha (ERα), and inhibit androgen synthesis, thereby disrupting ovarian hormone balance. For example, exposure to PFOS can reduce the acetylation level of histone H3K14 in the key steroidogenic enzyme, steroidogenic acute regulatory protein, within the ovaries, subsequently inhibiting estradiol synthesis and leading to follicular development disorders and ovulation defects (Feng et al., 2015). Animal studies have demonstrated that exposure to PFOA results in reduced progesterone levels, causing follicular development arrest (Landrigan et al., 2023), which aligns with the high-risk characteristics observed in infertile women in epidemiological studies. This hormonal imbalance may facilitate the development of ovarian cancer through disruption of the cell cycle or aberrant estrogen receptor (ER) signaling. A reduction in estradiol (E2) levels results in increased atresia and long-term accumulation of oxidative stress, which damages DNA and elevates the risk of gene mutations (Skrzypczak et al., 2024). PFAS may enhance the proliferation of ovarian cancer cells by activating ERα or inhibiting ERβ, thereby disrupting the ER α/β balance (Chang et al., 2023). In our study, estrogen receptor 1 (ESR1), also referred to as ERα, emerges as a critical target for both PFAS and ovarian cancer.

PFAS may contribute to the onset and progression of ovarian cancer by interfering with epigenetic mechanisms, including DNA methylation, histone modification, and microRNA expression. The mechanism of action may involve the silencing of key tumor suppressor genes, activation of oncogenic signaling pathways, and remodeling of the tumor microenvironment. PFAS may influence the epigenetic regulation of ovarian cancer by altering histone modifications (Lin et al., 2024). Specifically, histone deacetylation can lead to chromatin compaction and suppression of tumor suppressor gene expression. For example, elevated expression levels of HDAC1 have been linked to endometriosis, whereas aberrant HDAC activity in ovarian cancer may facilitate tumor progression by repressing the expression of tumor suppressor genes such as p16 (Terri et al., 2024). Concurrently, PFAS may modify the expression patterns of estrogen receptor-related genes by influencing the activity of histone-modifying enzymes, including H3K27me3 writing enzymes (Day et al., 2022). Endocrine disruptors can impact the expression of genes involved in steroid hormone synthesis and microRNAs through epigenetic regulation (Buck et al., 2011; Maxwell et al., 2024; Panwalkar et al., 2021), indicating that PFAS might disrupt ovarian hormone homeostasis via similar mechanisms, thereby indirectly promoting tumor development. In our study, HDAC1, HDAC2, and HDAC3 emerged as prioritized candidate mediators for both PFAS and ovarian cancer, suggesting that PFAS may influence the epigenetic regulation of ovarian cancer by altering histone modifications. Although our data suggest that HDACs may serve as key nodes linking PFAS exposure to ovarian cancer, we did not measure HDAC enzymatic activity or global histone acetylation levels. Such experiments are essential to confirm functional epigenetic disruption. Future experiments, including HDAC activity fluorometric assays and Western blotting for acetylated histone H3/H4, are needed. At the same time, PFAS may influence ECM remodeling by modulating the balance of the MMP system, potentially involving post-translational regulation of MMP9 activity. Future studies are needed by employing gelatin zymography and 3D culture models to assess MMP9 enzymatic function more accurately. Future research should integrate molecular biology experiments, animal models, and population cohorts to further elucidate the epigenetic toxicity mechanisms of PFAS and to explore intervention strategies targeting epigenetic pathways.

PFASs exert a profound influence on the metabolic reprogramming of ovarian cancer by disrupting oxidative phosphorylation, enhancing glycolysis, modulating lipid metabolism, and affecting epigenetic mechanisms (Hong et al., 2025). This impact is potentially mediated through the activation of the Warburg effect, inhibition of TFAM (transcription factor A, mitochondrial), and alteration of the tumor microenvironment. PFAS may associate with the Warburg effect, characterized by increased aerobic glycolysis, in ovarian cancer cells by impairing mitochondrial function (Hu et al., 2022). Studies have demonstrated that ovarian cancer cells preferentially utilize glycolysis over oxidative phosphorylation (OXPHOS) to rapidly produce ATP under conditions of hypoxia or metabolic stress. For instance, the reduced expression of TFAM is closely linked to metabolic reprogramming in platinum-resistant ovarian cancer cells (Gentric et al., 2019). TFAM plays a crucial role in maintaining mitochondrial function by regulating mitochondrial DNA replication and the expression of OXPHOS-related genes. Its diminished expression leads to the inhibition of OXPHOS, prompting a metabolic shift toward glycolysis, thereby enhancing resistance to platinum-based chemotherapeutic agents (Wculek et al., 2023). This phenomenon may be attributed to the metabolic interference caused by PFAS as these compounds can exacerbate the Warburg effect by inhibiting TFAM or by directly interfering with the activity of mitochondrial metabolic enzymes, such as complexes I and II. Future research should integrate multi-omics data, including metabolomics and epigenetics, to elucidate the mechanisms underlying the metabolic toxicity of PFAS. Additionally, it is imperative to explore intervention strategies aimed at metabolic reprogramming, such as the use of targeted TFAM or fatty acid synthase (FASN) inhibitors, to provide novel insights into the prevention and treatment of ovarian cancer.

As a persistent environmental pollutant, PFAS contributes to the pathogenesis of ovarian cancer through multiple pathways, including endocrine disruption, epigenetic modification, and metabolic reprogramming. Network toxicology offers an effective framework (Wu et al., 2024) for analyzing the ovarian toxicity mechanisms of PFAS by integrating multi-omics data and computational models. It is essential to employ prospective cohort studies and organoid models to verify the causality and identify intervention targets associated with PFAS and ovarian cancer, thereby providing a foundation for environmental policy development and clinical prevention strategies. The current experimental evidence is limited to PFOA; conclusions regarding other PFAS compounds remain inferential and require direct experimental confirmation. Moreover, ovarian granulosa cells are a primary target of PFAS toxicity, which can accumulate in follicular fluid, and KGN is a well-established *in vitro* model for studying PFAS effects on ovarian cells. Thus, KGN cells represent a granulosa-derived tumor model rather than the most common epithelial ovarian cancer subtype. The generalizability of our findings to epithelial ovarian cancer subtypes remains to be established through validation in relevant cell lines (SKOV3, OVCAR3, and A2780) and patient-derived models.

It is important to note that ovarian cancer is a multifactorial disease, and clinical samples are subject to confounding variables, including patients’ genetic backgrounds, disease stages, treatment histories, lifestyle factors, and diverse environmental exposures. These confounders complicated the attribution of changes in any single gene directly to exposure to PFOA. In contrast, the cell line qPCR data were derived from KGN cells exposed to acute PFOA treatment for 24 h *in vitro*, reflecting the short-term transcriptional response to direct chemical exposure. This study was designed to assess whether the target genes identified through network toxicology demonstrate direct transcriptional responses to PFAS exposure, thereby indicating the immediate adaptive responses of cells to chemical stimuli. The signaling pathways mediating PFOA-enhanced proliferation and migration remain to be elucidated. Molecular docking provides *in silico* predictions of binding affinities; however, these findings do not constitute evidence of direct target engagement or functional inhibition. Future studies employing SPR, ITC, and enzyme activity assays are required to validate these interactions Docking results should be interpreted as a basis for prioritizing candidates for further investigation, rather than as definitive evidence of binding or inhibition. Future mechanistic experiments including Western blotting for key signaling nodes (PI3K/AKT, MAPK/ERK, and TGF-β/SMADs) and the use of chemical inhibitors and siRNA knockdown are needed to establish functional causality.

This study has several limitations that must be acknowledged. First, network toxicology and molecular docking are inherently computational and hypothesis-generating tools; the 1,042 PFAS–protein interactions and docking results represent potential associations rather than experimentally validated physical interactions or evidence of functional inhibition and thus should be regarded as working hypotheses requiring validation through SPR, ITC, CETSA, and HDAC enzymatic activity assays. Meanwhile, DisGeNET curated genes and TCGA differentially expressed genes need to be validated further. Second, *in vitro* validation was limited to PFOA alone, yet different PFAS compounds vary significantly in carbon chain length and physicochemical properties, so conclusions regarding other PFAS compounds remain inferential and require systematic validation for PFOS, PFNA, and others. Third, the KGN cell line originates from granulosa cell tumors rather than epithelial ovarian cancer, and the generalizability of our findings to the predominant ovarian cancer subtype requires validation in SKOV3, OVCAR3, A2780, and patient-derived models. Fourth, although we observed PFOA-promoted proliferation and migration, the underlying signaling mechanisms remain to be elucidated; whether HDACs are essential mediators requires siRNA knockdown and inhibitor-based functional experiments, and HDAC enzymatic activity and histone acetylation levels were not measured, leaving the epigenetic claims without functional support. Fifth, *in vivo* animal models and patient cohort validation are completely lacking; the causal relationship between PFAS exposure and ovarian carcinogenesis requires prospective cohort studies and animal models, and candidate biomarkers need independent clinical validation. Finally, mRNA expression changes reflect transcriptional responses; however, corresponding protein-level changes were not assessed, and clinical tissue data reflect long-term steady-state expression, whereas KGN qPCR data reflect short-term acute transcriptional responses; these datasets originate from distinct experimental systems and should be interpreted independently. In summary, this study provides an integrative predictive framework and prioritized candidate targets for understanding PFAS-associated ovarian carcinogenesis; the limitations outlined above define the experimental roadmap required to translate these predictions into mechanistically confirmed conclusions, and we invite the scientific community to test our predictions through independent approaches.

In conclusion, we employed an integrative approach combining network toxicology, protein interactions, and molecular docking to identify multiple potential targets associated with ovarian cancer. It systematically elucidated the mechanistic connections between PFAS and ovarian cancer, offering novel insights for toxicology and environmental health research. This approach also paves the way for further exploration of the impact of various pollutants on human diseases. The findings suggest that eight key genes, HDAC1, SIRT1, ESR1, RXRA, PTGS2, MMP9, HDAC2, and HDAC3 may represent candidate biomarkers and potential therapeutic targets, but these proposals require rigorous clinical validation. These results provide valuable theoretical insights into the molecular mechanisms by which PFASs influence ovarian cancer progression and identify promising molecular targets for early detection, prognostic assessment, and targeted therapy of ovarian cancer. We anticipate that these findings will stimulate further research into the environmental factors contributing to ovarian cancer and foster the development of more effective prevention and treatment strategies. It is important to emphasize that the mechanistic interpretations presented in this study are hypothesis-generating and require rigorous experimental validation. The computational predictions and preliminary *in vitro* observations should serve as a foundation for future hypothesis-driven research rather than as definitive conclusions. Future studies should systematically investigate multiple PFAS compounds across a panel of ovarian cancer cell models. We hope that this study will resonate globally, underscoring the imperative for international collaboration to reduce the prevalence of PFAS and mitigate their impacts on ecosystems and public health through coordinated efforts.

## Data Availability

The raw data supporting the conclusions of this article will be made available by the authors, without undue reservation.

## Supplementary material

SUPPLEMENTARY FIGURE S1 Flowchart for target selection of PFAS in ovarian cancer.
