# Supplementary material for: Integrative network toxicology and molecular docking reveal epigenetic and endocrine-disrupting mechanisms of PFAS in ovarian cancer
Source: Front Pharmacol. 2026 Aug 28;17:1905013. doi: 10.3389/fphar.2026.1905013 (PMC13561819; doi:10.3389/fphar.2026.1905013)
Supplement: Supplementary file 1 [file Image1.pdf]

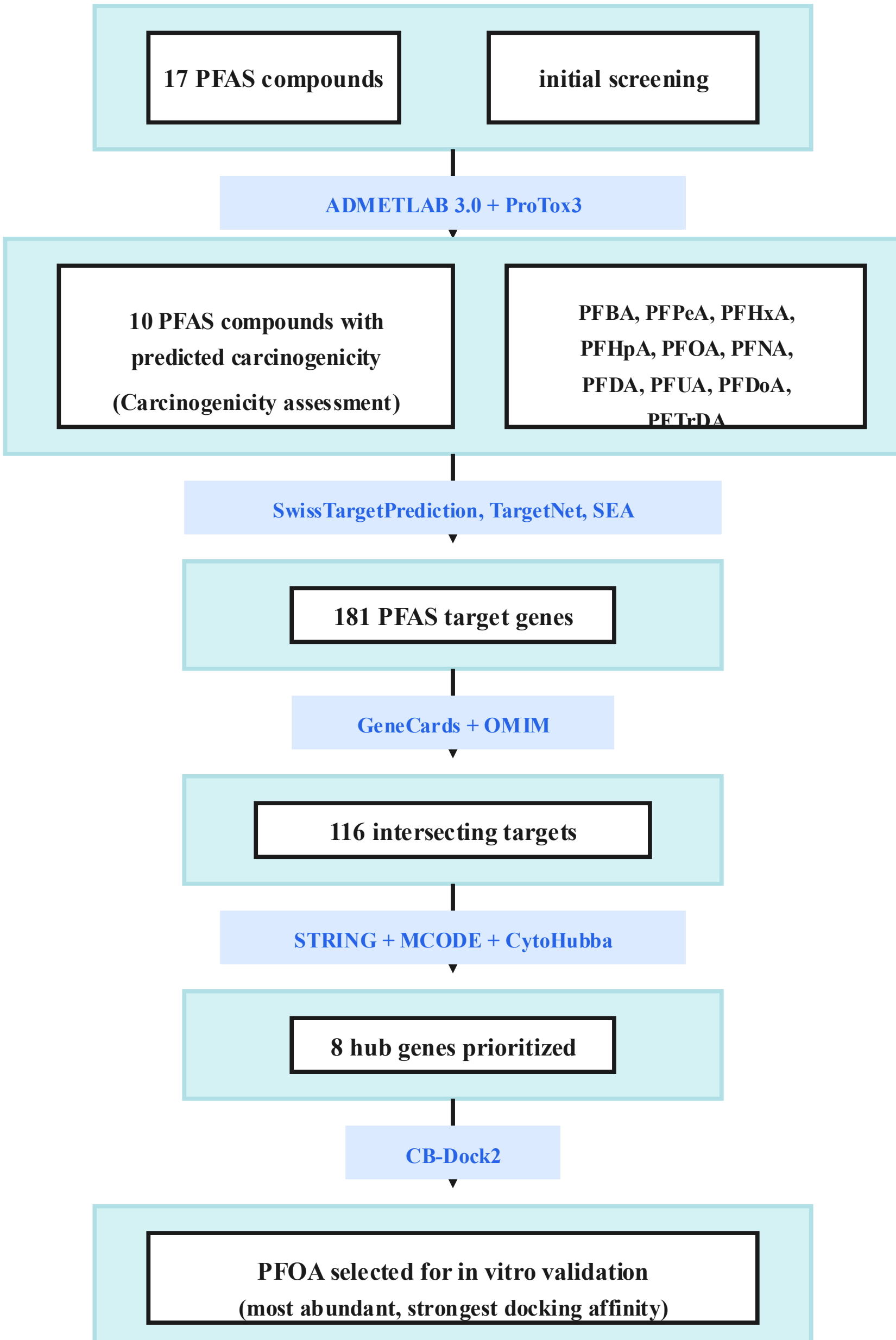
17 PFAS compounds
initial screening
ADMETLAB 3.0 + ProTox3
10 PFAS compounds with predicted carcinogenicity
(Carcinogenicity assessment)
PFBA, PFPeA, PFHxA, PFHpA, PFOA, PFNA, PFDA, PFUA, PFDoA, PFTrDA
SwissTargetPrediction, TargetNet, SEA
181 PFAS target genes
GeneCards + OMIM
116 intersecting targets
STRING + MCODE + CytoHubba
8 hub genes prioritized
CB-Dock2
PFOA selected for in vitro validation
(most abundant, strongest docking affinity)

Figure_S1
